# Two-step intensity modulated arc therapy (2-step IMAT) with segment weight and width optimization

**DOI:** 10.1186/1748-717X-6-57

**Published:** 2011-06-02

**Authors:** Jidi Sun, Theam Yong Chew, Juergen Meyer

**Affiliations:** 1 University of Canterbury, Department of Physics & Astronomy, Private Bag 4800, Christchurch 8140, New Zealand; 2 Lincoln Ventures Ltd, Engineering Drive, Lincoln University, Christchurch 7640, New Zealand

## Abstract

**Background:**

2-step intensity modulated arc therapy (IMAT) is a simplified IMAT technique which delivers the treatment over typically two continuous gantry rotations. The aim of this work was to implement the technique into a computerized treatment planning system and to develop an approach to optimize the segment weights and widths.

**Methods:**

2-step IMAT was implemented into the Prism treatment planning system. A graphical user interface was developed to generate the plan segments automatically based on the anatomy in the beam's-eye-view. The segment weights and widths of 2-step IMAT plans were subsequently determined in Matlab using a dose-volume based optimization process. The implementation was tested on a geometric phantom with a horseshoe shaped target volume and then applied to a clinical paraspinal tumour case.

**Results:**

The phantom study verified the correctness of the implementation and showed a considerable improvement over a non-modulated arc. Further improvements in the target dose uniformity after the optimization of 2-step IMAT plans were observed for both the phantom and clinical cases. For the clinical case, optimizing the segment weights and widths reduced the maximum dose from 114% of the prescribed dose to 107% and increased the minimum dose from 87% to 97%. This resulted in an improvement in the homogeneity index of the target dose for the clinical case from 1.31 to 1.11. Additionally, the high dose volume V_105 _was reduced from 57% to 7% while the maximum dose in the organ-at-risk was decreased by 2%.

**Conclusions:**

The intuitive and automatic planning process implemented in this study increases the prospect of the practical use of 2-step IMAT. This work has shown that 2-step IMAT is a viable technique able to achieve highly conformal plans for concave target volumes with the optimization of the segment weights and widths. Future work will include planning comparisons of the 2-step IMAT implementation with fixed gantry intensity modulated radiotherapy (IMRT) and commercial IMAT implementations.

## Background

Intensity modulated-arc therapy (IMAT) is an advanced form of intensity modulated radiation therapy (IMRT) [1]. IMAT was first introduced by Yu [2] as a rotational treatment technique which irradiates the target during gantry rotation as opposed to utilizing fixed gantry angles for IMRT. Since Yu's seminal paper in 1995, several approaches to IMAT have been described in the literature [3-5]. Pioneering work was based on in-house implementations and therefore limited to research institutions. With the availability of commercial solutions, such as Elekta's (Elekta Ltd, Crawley, UK) Volumetric Modulated Arc Therapy (VMAT) and Varian's (Varian Medical Systems, Palo Alto, CA) RapidArc^®^, IMAT has the potential to become the method of choice for complex cases for many radiation oncology facilities. While the dosimetric benefits of IMAT over IMRT have been analyzed and debated in numerous publications [6-9] the clinical outcomes have yet to be published. The main advantage of IMAT is thought to be from a health economic perspective. Despite the increased complexity of IMAT, most studies have indicated that the actual treatment times on the linear accelerator (linac) are shorter than for conventional IMRT [3,10-13]. This brings several prospective advantages such as reduced probability of patient/organ movement, more time for image guidance and a reduced chance of the loss of biological effectiveness [14-16]. From an administrative point of view, the promise is that this will allow more patients to be treated per day on a given linac and therefore increase patient throughput. However, as the transition from conventional 3D conformal radiotherapy (3DCRT) to IMRT has shown, a more complex technique puts a heavy burden on departments [17,18]. When comparing fixed gantry IMRT with IMAT, the increased complexity will, at least initially, most likely also result in increased planning times [13] and more stringent QA and patient specific verification procedures. With regard to the latter, non-intensity modulated 3DCRT treatments only require machine specific QA. Intensity modulated techniques on the other hand require patient specific QA [19] due to the number and complexity of the non-intuitive shapes of the beam segments. An additional level of complexity is added when going from fixed gantry IMRT to IMAT due to the dynamic nature of the treatment. Not only does the gantry rotate during delivery, the individual multileaf collimator (MLC) leaves, and depending on the approach chosen, the dose rate, gantry speed, collimator angle and couch motion [20] may also vary. To achieve this, sophisticated hardware and software is required and many existing linacs cannot deliver such a treatment [21].

A simplified approach to intensity modulated arc therapy for concave target volumes is 2-step IMAT. 2 step-IMAT aims to reduce the aforementioned complexity in planning, QA, verification and delivery by taking advantage of the geometrical relationship and more intuitive beam segments. 2-step IMAT was proposed by Bratengeier [22] and is based on Brahme's original work in the 1980's [23,24]. Brahme *et al*. used a physical non-linear wedge filter to shape the intensity of the incident beam onto a cylindrical ring shaped planning target volume (PTV). The purpose of the filter was to create a non-uniform beam intensity profile in order to improve the dose uniformity inside the PTV. The significance of Brahme *et al*.'s work was that the resulting ideal continuous intensity profile was high in intensity close to the organ-at-risk (OAR) and continuously tapered off away from the OAR. With this deliberate intensity modulation the dose gradient between the PTV and adjacent OAR was increased considerably and the dose uniformity within the PTV improved.

The fundamental idea of 2-step IMAT is to approximate the ideal intensity profile, referred to by Brahme, with two discrete intensity levels created by means of two non-modulated beam apertures, henceforth referred to as the 1^st ^and 2^nd ^order segments. Bratengeier *et al*. have successfully applied this approach to phantoms and clinical cases with concave PTVs for both fixed gantry angles (2-step IMRT) [25,26] and rotational irradiation (2-step IMAT). It was demonstrated that the resulting plans were comparable or even superior to conventional IMRT plans [25]. The complexity of these 2-step plans was kept to a minimum, as reflected in the small number of segments for 2-step IMRT, the intuitive shapes of the beam segments and the minimal MLC movement from one gantry angle to another for 2-step IMAT. 2-step IMRT has also shown great promise with regard to online adaptive radiotherapy due to the geometric relationship between organs and beam segments [27,28].

To date, the 2-step technique has not been implemented into a computerized treatment planning system. Although the 2-step IMRT technique has been successfully applied clinically by Bratengeier *et al*., the beam segment generation was performed manually in a commercial treatment planning system with consecutive optimization of the segment weights and shapes [26]. The manual generation of 2-step IMAT plans would require many segments to be generated by hand, which makes it impractical and prohibitive for clinical use. This work implements 2-step IMAT into a computerized treatment planning system. The implementation consists of automatic beam segment generation and consecutive dose-volume based plan optimization in analogy to inverse planning. It should be noted that the aim of this work was neither to investigate the suitability of the 2-step IMAT technique for different treatment sites nor as an alternative to other IMAT techniques. The main focus is on the actual implementation and associated optimization.

## Methods

2-step IMAT was implemented into the current version (Version 1.51) of the University of Washington treatment planning system Prism [29-32]. Prism is written in Common Lisp; the source code is freely available for non-commercial use. Prism has been in clinical use since 1994 and has full 3DCRT planning capabilities. It was chosen for the implementation because it allows additional Lisp code to be loaded during runtime. This makes it convenient to modify and add features to Prism [30,33]. In the following subsection, the implementation of 2-step IMAT into Prism is described. This is followed by the application of the implemented approach to a phantom and a clinical case. It is noted that in this work the technicalities of the actual delivery of the 2-step IMAT plans on a linac are not explicitly addressed but will be briefly discussed in the Results and Discussion section.

### Implementation

#### Segment generation

**Figure 1:**
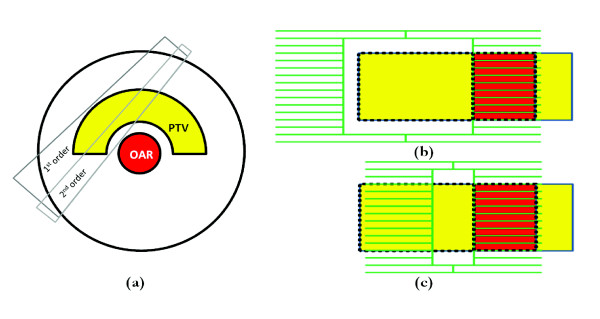
**Illustration of the phantom and the 2-step IMAT segment generation**. (a) Transverse view and (b) and (c) BEV. The 1^st ^order segment is shown in (b), the 2^nd ^order narrow segment in (c).

2-step IMAT is delivered in two continuous gantry rotations. Each rotation consists of a sequence of control points, henceforth referred to as beam segments. A 2-step IMAT treatment plan therefore possesses two beam segments at each gantry angle [22]. The 1^st ^order segments cover the PTV in the beam's-eye-view (BEV), excluding the volume overlapping with the OAR. The 2^nd ^order segments are narrow segments adjacent to the OAR in the PTV. This is illustrated in Figure 1. Both the 1^st ^and 2^nd ^order beam segments are shaped in the beam's-eye-view (BEV) based on the geometry of the PTV and OAR. At each gantry angle, the 3D point clouds that form the structure contours are projected onto a 2D plane perpendicular to the central axis through the isocentre [34]. The outermost points of the projection of an organ constitute the outline of that particular organ on the plane. All the projected organ outlines are superimposed onto the plane and thus provide information on the positions of various organs in the BEV. For certain geometries, there are two regions of the PTV (on either side of the OAR) that qualify for portal shaping in the BEV [7]. Ideally one wants to irradiate both regions at the same time to maintain the efficiency and quality of the plan, but this attempt is limited by the physical limitation of the MLC leaves. Therefore, the radiation may only be delivered to one part of the PTV region during one continuous gantry rotation to minimize the movement of the MLC. In the current implementation, if segments are found on either side of the OAR during the segment generation process, only the segment on the pre-selected side (left or right) is kept. This applies to both order segments. An illustration of the segment generation implemented in this work is shown in Figure 1. Note that in this example the segments on the left side of the OAR are shown in the BEV. At certain gantry angles, in this example, in the region around 270°, no segments can be generated on the left of the OAR. Consequently, the MLC leaves are closed and the monitor units set to zero and excluded from the optimization process later on.

**Figure 2:**
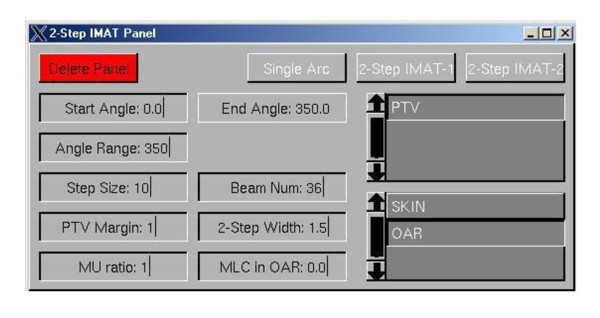
**Screenshot of the 2-step IMAT GUI**.

To reiterate, delivery of the treatment is by means of two rotations, each of which comprises the segments of each order. The implementation also includes a margin around the PTV for MLC positioning of the 1^st ^order segment, i.e. margins in superior-inferior direction as well as in lateral direction, in order to compensate for the dose fall-off at the beam edges due to the penumbra [35]. For ease of operation, a graphical user interface (GUI) was created to allow the treatment planner to enter the necessary set-up parameters for the automatic generation of the 2-step IMAT beam segments. The GUI is shown in Figure 2.

#### Beam segment weight optimization

Once all *n *beam segments have been automatically generated in Prism, each segment is initially allocated a unity beam weight *x_i _*= 1, with *i *= 1... *n*. A variable dose grid was implemented for efficiency so that finer point spacing could be used for dose point sampling in smaller organs, such as e.g. the spinal cord, while a coarse dose grid can be used for larger organs, such as e.g. the lung and liver. The dose points *d_j_*, with *j *= 1... *p*, as distributed on the grid, were calculated using(1)

or in matrix notation(2)

The matrix **M **is calculated by the Prism dose engine [36] and consists of all the contributions *m_ji _*of the beam segments *i *to the dose points *j*. Each element in the row of matrix **M **contains the contribution of all the segments to a single point and each element in the column of the matrix **M **contains the contribution of a single beam to every dose point. Matrix **M **is considered to be a constant so a desired dose distribution can be obtained by altering the beam segment weights **x**, which represent linac monitor units (MUs). In this work, the optimization of the beam segment weights, **x**, was implemented in Matlab R2009a (The MathWorks, Inc., Natick, MA, USA) with a dose-volume (DV) based quadratic objective function [37,38] in combination with *fmincon*, an inbuilt constrained non-linear optimization search method [39]. The lower constraint boundaries were set to zero segment weight. The upper limit MU constraint can be adjusted to the specific capabilities of a particular linac and was set to a value of 10 MUs in order to ensure that individual weights would not become unreasonably high.

The individual objective function terms, or costlets, *c_r_*, are given by:(3)

where

For each dose-volume objective, the costlet, *c_r_*, is represented by the multiple of an assigned weighting factor, *w_r_*, and the sum of squared difference between each point dose, *d_j_*, and the dose objective, *d_obj_*, times the conditional term ψ and divided by the number of dose points, *p*. The dose *d' *corresponds to the intersection of the horizontal connection between the DV objective point (with dose *d_obj _*and volume *v_obj_*) with the DVH curve. The Heaviside function, *H*, is used to select from different types of DV objectives for the cost calculation with

The *maximum DV objective *is a planning objective used to minimize irradiation of OARs and reduce PTV hot spots. The *minimum DV objective *is used to penalize cold spots in the PTV. The composite cost, *C*, for all *l *individual objective terms is given by:(4)

with the optimization goal: min(*C*(**x**)).

**Figure 3:**
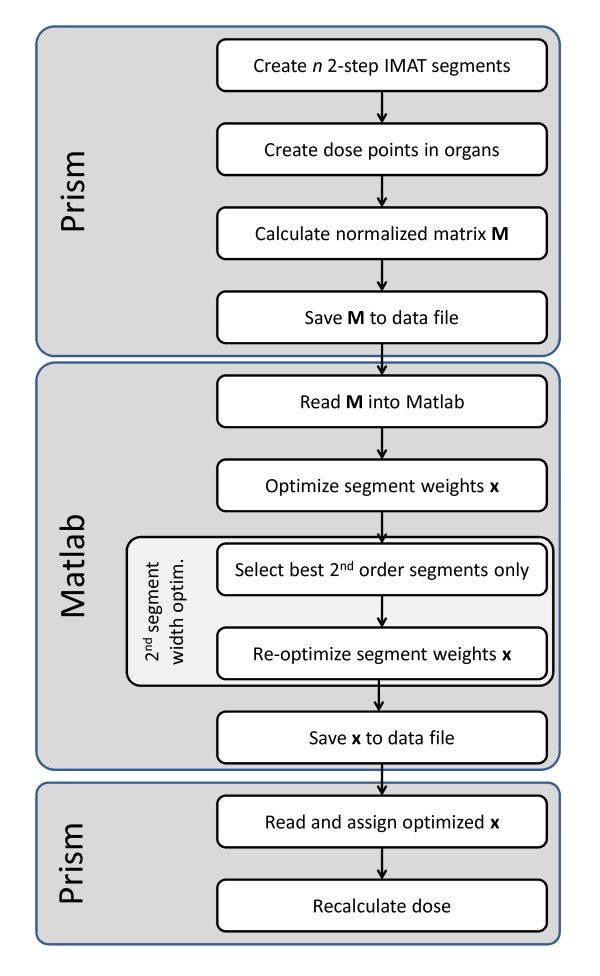
**Flowchart illustrating the workflow of the 2-step IMAT implementation**.

Once the optimized beam weights had been determined, they were imported back into Prism. The final dose distribution was recalculated using the Prism dose engine based on a macro pencil beam model [40]. The overall workflow of the implementation is summarized in Figure 3.

### Phantom

The 2-step IMAT implementation was first applied to a virtual cylindrical phantom with unit density. The phantom (diameter ø = 30 cm) has been used previously by Bratengeier [22] and consists of a horseshoe-shaped PTV (ø_inner _= 8 cm, ø_outer _= 20 cm) wrapped around a cylindrical OAR (ø = 6 cm) as illustrated in Figure 1a. A systematic sensitivity analysis was carried out to determine the optimal parameters in terms of dose grid size, number of discrete gantry angles to simulate rotational irradiation, 2^nd ^order segment width, margins, speed of the optimization and quality of the plan. The details of the sensitivity analysis are beyond the scope of this paper and are described elsewhere [41]. However, one of the findings of this analysis was that a beam angle spacing of 5°constitutes an adequate representation of a rotational treatment. For the optimization procedure, the dose point sampling space was 0.7 cm for the PTV and 0.3 cm for the OAR. An Elekta SL linac from the Prism database was utilized, with a 6 MV beam and an MLC with 40 leave pairs, projecting to 1 cm at isocentre. A 1 cm margin around the PTV was applied for MLC positioning for the 1^st ^order beam segments in all directions except for the boundary close to the OAR. The margin was chosen to minimize the effects of the beam penumbra on PTV dose uniformity [41].

To verify the implementation a comparative planning study was carried out using the following treatment planning strategies:

Plan 1. *One full *rotation with 1^st ^order segments only, segment weight optimized (corresponds to an optimized conformal arc).

Plan 2. *Two full *rotations with 1^st ^order and *fixed width *2^nd ^order segments, width of 2^nd ^order segment was 1.5 cm, segment weight optimization.

Plan 3. *Four full *rotations with 1^st ^order and three different *fixed width *2^nd ^order segments, width of 2^nd ^order segments were 1 cm, 1.5 cm and 2 cm, segment weight optimization.

Plan 4. The same as plan 3 except that only the highest weighted 2^nd ^order segment per gantry angle was selected and the other 2^nd ^order segments from this gantry angle were deleted so that the plan could be delivered with *two full *rotations. The weights were then re-optimized.

It is noted that a fixed width 2^nd ^order segment plan (Plan 2) is not optimal but served as a reference for individualized width optimization for each gantry angle (Plan 4). In previous work [41], plans with different fixed width 2^nd ^order segments were compared and a width of 1.5 cm was found to be the most favourable in terms of the homogeneity index (maximum PTV dose divided by minimum PTV dose) for the given phantom geometry. For more complex geometries it might be beneficial to vary the width of the 2^nd ^order segments from one gantry angle to another but also to vary the gap and position of individual leave pairs within the 2^nd ^order segment. Ideally, the individual leaf positions for the 2^nd ^order segment should be optimized from each direction. An approximation of the ideal 2^nd ^segment shape can be found by generating multiple 2^nd ^order segments of different width (Plan 3) to give the optimization more degrees of freedom to find a better solution. As this results in four full rotations, only the 2^nd ^order segment with the highest weight per gantry angle were selected in Plan 4 to reduce the number of gantry rotations. The aim was to investigate whether this straightforward 2^nd ^order segment width optimization could provide an improvement in PTV dose uniformity over fixed width 2^nd ^order segments (Plan 2).

To avoid user bias, all plans were optimized using the same objectives. The objectives of the optimization for the PTV were to deliver at least 97% of the prescribed dose to at least 96% of the PTV volume. No more than 2% of the PTV volume should receive more than 105% of the prescribed dose. The sole OAR objective was to deliver no more than 41% of the prescribed dose to more than 1% of the OAR volume. The weighting factors for the above three objectives were 10, 5, and 1, respectively. After the optimization was complete, all plans were normalized to D_95 _and the homogeneity index calculated for the final comparison.

### Clinical case

To test the implementation on a clinical case, the data of a paraspinal tumour patient treated at the University of Wuerzburg were selected. The DICOM CT data and radiotherapy structure sets were imported into Prism. The non-symmetrical target volume was in close proximity to the spinal cord and wrapped around the critical structure. The cross-section of the PTV along the longitudinal direction varied and the axis of the spinal cord was tilted by approximately 8°with respect to the patient axis. The dose objective for this planning study was to deliver 60 Gy (corresponding to 100%) to the target volume and a maximum of 40 Gy (corresponding to 67%) to the spinal cord. A secondary objective was to keep the dose to the lungs and liver at a minimum. The grid size for the sampling of the PTV and the spinal cord were set to 0.2 cm and 0.1 cm, respectively, resulting in 3064 and 2354 dose points uniformly distributed inside the two volumes.

Three 2-step IMAT plans were generated for this clinical case:

Reference Plan 5 consisted of 1^st ^order segments with a 0.5 cm margin around the PTV for MLC positioning and a fixed 2^nd ^order segment width of 1.0 cm at all gantry angles. Analogous to the phantom case, several plans were previously compared with different fixed 2^nd ^order segment widths [41] for this clinical case. A width of 1 cm resulted in the best homogeneity index and was therefore chosen for the reference plan.

Plan 6 consisted of the same 1^st ^order segments plus three different 2^nd ^order beam segment widths (0.5 cm, 1.0 cm and 1.5 cm). The widths were chosen to cover the most likely range based on previous findings [41-43]. The segment weights of Plan 5 and 6 were then individually optimized in Matlab using the following objectives. The PTV was to receive at least 98% of the prescribed dose to 98% of the volume, and no more than 3% of volume should receive more than 105% of the prescribed dose. The OAR should receive no more than 60% of the prescribed dose. The weighting factors of the above objectives were 100, 70 and 20, respectively.

Based on the optimized result of Plan 6, only the highest 2^nd ^order segment amongst the three 2^nd ^order segments from each gantry angle were selected for Plan 7. The final step was to re-optimize the segment weights for Plan 7 using the same objectives as before.

## Results and Discussion

### Phantom Study

**Figure 4:**
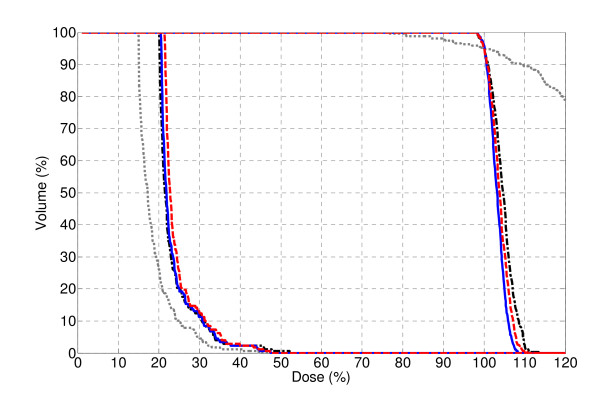
**Dose volume histogram of Plan 1 (gray dot), Plan 2 (black dash-dot), Plan 3 (blue solid) and Plan 4 (red dash) for the phantom**. All plans were normalized to D_95 _= 100%.

The dose-volume histograms (DVH) for plans 1-4 are shown in Figure 4. Although Plan 1 was able to minimize OAR irradiation, the uniformity of the target coverage was greatly affected by the lack of intensity modulation. The minimum and maximum dose were 76% and 166%, respectively, and the homogeneity index, a measure of the uniformity of the PTV dose distribution, was 2.18 (see Table 1), illustrating the lack of uniformity of Plan 1. This proof-of-principle result confirmed the findings by Brahme *et al*. on the necessity of certain intensity modulation for complex geometries in order to achieve a uniform and conformal dose.

**Table 1:** Comparison of the plan results for the phantom.

	Plan 1	Plan 2	Plan 3	Plan 4
**D_PTV, max _(%)**	165.9	113.4	108.4	110.7

**D_PTV, min _(%)**	76.1	97.6	97.6	97.4

**V_107, PTV _(%)**	90.6	20.7	3.3	12.3

**HI**	2.18	1.16	1.11	1.14

**D_OAR, max _(%)**	44.6	52.2	47.5	47.8

Of Plans 2-4, Plan 3 achieved the best PTV dose uniformity. This can be attributed to the increased number of segments and therefore gantry rotations. Both Plan 2 and 4 utilize only one 2^nd ^order segment at each gantry angle, therefore the treatment can be delivered with two gantry rotations. Due to the reduced number of segments, a slight trade-off can be observed for Plan 2 and 4 in terms of the PTV dose uniformity and maximum OAR dose with regard to Plan 3. Plan 4 achieved a more uniform PTV dose coverage than Plan 2, which used a constant 2^nd ^order segment width.

**Figure 5:**
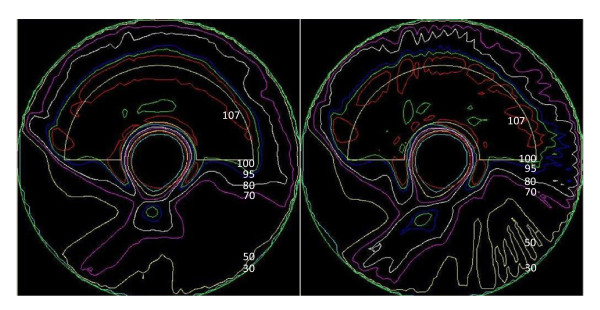
**Dose distribution comparison for the phantom between (a) Plan 2 and (b) Plan 4**. Isodose lines: 107 (red), 100 (green), 95 (blue), 80 (white), 70 (purple), 50 (yellow), 30 (cyan).

Figure 5 compares the dose distributions of Plan 2 and Plan 4 in the central transverse plane. It can be seen that the 95% isodose line wraps conformally around the PTV, while sparing the OAR. Plan 4 reduced the hot spot region in the PTV when compared with Plan 2. Note that for simplicity, no dose constraint was used for the body. The maximum dose outside the PTV was 112% for Plan 4.

This phantom study verified the efficacy of the implementation and demonstrated that the implemented 2^nd ^segment width optimization can indeed improve the plan quality without increasing the complexity. In fact, when choosing the isocentre conveniently, such that it is in the centre of the inner radius of the target, the inner MLC leaf bank remains more or less stationary, shadowing the OAR throughout each rotation. The outer leaf bank moves only minimally, depending on the geometry of the PTV for the 1^st ^order segment, and the range of widths included in the optimization for the 2^nd ^order segments (1 cm in this case).

### Clinical Case

**Figure 6:**
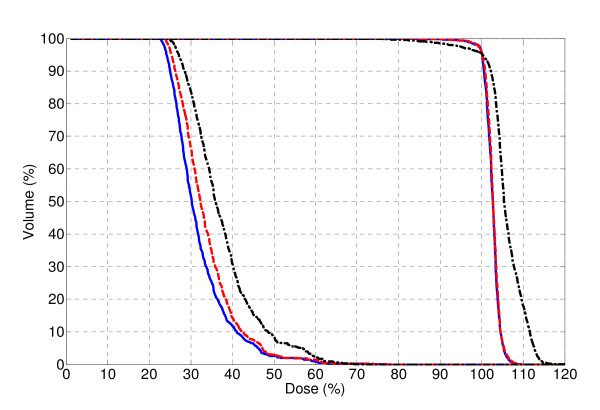
**PTV and spinal cord DVH comparison of Plan 5 (black dash-dot), Plan 6 (blue solid) and Plan 7 (red dash)**. All plans were normalized to D_95 _= 100%.

**Figure 7:**
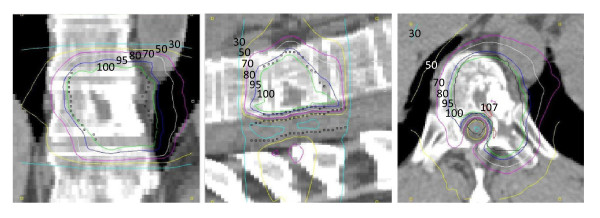
**Coronal, sagittal and transverse dose distribution of Plan 7 for the clinical case**. PTV and OAR contours are black. Isodose line: 107 (red), 100 (green), 95 (blue), 80 (white), 70 (purple), 50 (yellow), 30 (cyan).

The DVH comparison in Figure 6 illustrates the benefits of 2^nd ^order segment width optimization. The results show the same trend as for the phantom case. An obvious improvement in PTV uniformity can be seen when comparing Plan 5 with Plan 6. The initial objective of a homogeneous dose distribution corresponding to 60 Gy in the PTV and a maximum dose of 40 Gy, corresponding to 67%, in the OAR could clearly be achieved. The DVH for the PTV is almost identical for Plans 6 and 7, while the dose to the spinal cord is somewhere between that of Plans 5 and 6. The isodose distribution in the three cardinal cross-sections for Plan 7 is shown in Figure 7. The quality of the plans is further quantified in Table 2, where D_1 _and D_99 _correspond to the maximum and minimum dose respectively, and V_105 _corresponds to the volume receiving more than 105% of the dose. The composite objective value after optimization is represented by *C*(**x**)

**Table 2:** Comparison of the plan results for the clinical case.

	Plan 5	Plan 6	Plan 7
**D_1, PTV _(%)**	114.4	106.9	107.2

**D_99, PTV _(%)**	87.1	96.7	97.2

**V_105, PTV _(%)**	56.6	6.3	7.0

**HI**	1.31	1.11	1.10

**D_1, OAR _(%)**	62.4	59.5	60.5

*C*(**x**)	611.65	10.61	11.41

Plan 6 resulted in the best plan among the three plans, but four continuous rotations are necessary to deliver it. This would counteract one of the advantages of 2-step IMAT, which is to reduce the complexity of the plan. Conversely, Plans 5 and 7 consist of only one 2^nd ^order segment per gantry angle, so two gantry rotations are sufficient to deliver the plan. With only half the number of segments, Plan 7 was able to achieve virtually the same PTV dose uniformity of HI = 1.1 as Plan 6, while keeping the OAR dose at a similar level.

The results obtained for the spinal case are encouraging. There is further potential for improvement by optimizing the segment widths in smaller increments over a wider range or even each individual leaf, similar to the work by Claus *et al*. for forward planned IMRT [44] and others [4,45,46]. The trade-off however would be a significant increase in optimization time due to the large number of variables that would have to be optimized and the fact that because of the myriad of different MLC constellations, pre-calculation of the dose matrices would be infeasible within a practical time frame. The straightforward approach presented here is efficient. The segment generation in Prism generally took less than one minute on an Intel dual core CPU with 2.66 GHz and 1 GB RAM running Red Hat release 5.1.Segment weight optimization in Matlab took approximately 10 min for the clinical case. The latter can potentially be sped up by implementing the optimization in Common Lisp within Prism and by using alternative optimizations methods such as projection-onto-convex sets (POCS), which has been implemented in Prism for IMRT optimization [47,48].

In terms of the actual plan delivery, 2-step IMAT plans with variable segment weights require a linac capable of variable dose rate delivery and/or variable gantry speed. For example, to deliver a dose of 2 Gy for the paraspinal case a mean dose rate of 1.8 ± 0.8 MUs/degree (1 SD) would have been necessary. This indicates that no drastic variations in dose rate would be required for this particular case. Tang *et al*. have recently proposed an approach to deliver IMAT plans on a standard linac with constant dose rate by redistributing the segment weights (corresponding to a constant arc length) to unevenly spaced angular intervals such that the segments with larger MU weighting occupy a greater angular length [21]. This approach is based on the fact that rotational delivery is not sensitive to small angular deviations. The same approach should theoretically be possible with 2-step IMAT plans and paves the way for the delivery of 2-step IMAT on standard linacs without variable dose rates.

In this work no linac specific delivery constraints were included in the optimization. Including the IMAT delivery constraints would ensure that the plan is deliverable [49]. For the optimized paraspinal tumour plan (Plan 7) the maximum motion between 2^nd ^order beam segments may be as much as 1 cm, corresponding to a segment width between 0.5 and 1.5 cm. To estimate whether delivery of this plan would be feasible the following machine constraints for a Varian linac were taken from the literature. Assuming a maximum gantry speed of 4.8°/s and a maximum leaf speed of 2.25 cm/s the maximum permitted leaf motion would be 0.47 cm/° [50]. For a 5°spacing between control points this would result in maximum permitted MLC leaf motion between control points of 2.35 cm. The maximum MLC motion for Plan 7 is 1 cm, well within the limits of current linac capabilities.

An area for further work would also be to investigate the feasibility of delivering a 2-step IMAT plan in one rotation by alternating between the 1^st ^and 2^nd ^order segments. This would require that the linac hardware constraints are taken into account in the optimization process.

## Conclusions

2-step IMAT has been successfully implemented into a computerized treatment planning system by automatically generating the MLC segments in the BEV. The optimization of the weights and the widths of the 2^nd ^order segments were carried out using Matlab. The automatic generation of the MLC segments makes it possible to apply 2-step IMAT to more clinical cases, which has so far been tedious as the segments had to be generated manually.

The phantom study illustrated the benefits of 2-step IMAT over a conventional single optimized non-modulated arc technique and demonstrated the feasibility of 2-step IMAT with the current implementation. The intensity modulation achieved by delivering two discrete and uniform segments to produce a simple 2-step intensity modulation considerably improved the dose uniformity of the PTV while keeping the dose to critical organs to a minimum. By optimizing the weights and widths of the 2^nd ^order segments, the quality of the plans could be improved with regard to both PTV uniformity and OAR sparing. This improvement was also observed for the clinical paraspinal tumour case.

The results have shown that plan generation can be simplified using the prior knowledge of the relationship between the geometry of the anatomy and the corresponding intensity modulation. This planning study has shown that 2-step IMAT lends itself well for paraspinal tumours where high dose gradients close to the OAR are required. Furthermore, Bratengeier *et al*. have shown that it is possible to apply 2-step IMAT to cases with multiple OARs [42] and also simultaneous integrated boosts [51]. The current implementation can only handle one PTV and one OAR. The automation of 2-step IMAT planning for multiple OARs remains an area for further work.

It should be emphasized that 2-step IMAT is not only less complex than more sophisticated IMAT techniques, it also puts less demand on the linac and MLC leaves due to minimal changes in the field shape from one gantry angle to another. Moreover it can potentially be delivered on a linac without variable dose rates. This would have positive ramifications in terms of linac maintenance and QA.

In terms of future work, a rigorous comparison between the commercial implementation of fixed gantry IMRT, IMAT and 2-step IMAT for different treatments sites is required to fully quantify the overall benefits and trade-offs of the described approach. For this to be relevant, the linac specific delivery constraints must be taken into account.

## Competing interests

The authors declare that they have no competing interests.

## Authors' contributions

JS conducted the main part of the work as part of his MSc thesis in Medical Physics.

TYC was involved in the implementation and optimization part of the approach. He also contributed significantly to the drafting and reviewing of the manuscript. JM initiated the research and came up with the conceptual idea. He contributed significantly to the drafting and reviewing of the manuscript. All authors have read and approved the final manuscript.
